# The Next-Generation Immune Checkpoint LAG-3 and Its Therapeutic Potential in Oncology: Third Time’s a Charm

**DOI:** 10.3390/ijms22010075

**Published:** 2020-12-23

**Authors:** Quentin Lecocq, Marleen Keyaerts, Nick Devoogdt, Karine Breckpot

**Affiliations:** 1 Laboratory for Molecular and Cellular Therapy (LMCT), Vrije Universiteit Brussel, Laarbeeklaan 103, B-1090 Brussels, Belgium; quentin.lecocq@vub.be; 2 Nuclear Medicine Department, UZ Brussel, Laarbeeklaan 101, B-1090 Brussels, Belgium; Marleen.Keyaerts@vub.be; 3 In Vivo Cellular and Molecular Imaging Laboratory (ICMI), Vrije Universiteit Brussel, Laarbeeklaan 103, B-1090 Brussels, Belgium; ndevoogd@vub.be

**Keywords:** LAG-3, immune checkpoint, immunotherapy, diagnosis, cancer, oncology

## Abstract

The blockade of immune checkpoints (ICPs), such as cytotoxic T lymphocyte associated protein-4 (CTLA-4) and programmed death-1 (PD-1) and its ligand (PD-L1), has propelled the field of immuno-oncology into its current era. Drugs targeting these ICPs have improved clinical outcome in a number of patients with solid and hematological cancers. Nonetheless, some patients have no benefit from these ICP-blocking therapies. This observation has instigated research into alternative pathways that are responsible for the escape of cancer cells from anti-cancer immune responses. From this research, a number of molecules have emerged as promising therapeutic targets, including lymphocyte activating gene-3 (LAG-3), a next-generation ICP. We will review the current knowledge on the biological activity of LAG-3 and linked herewith its expression on activated immune cells. Moreover, we will discuss the prognostic value of LAG-3 and how LAG-3 expression in tumors can be monitored, which is an aspect that is of utmost importance, as the blockade of LAG-3 is actively pursued in clinical trials.

## 1. Introduction

Immune cells are controlled by a plethora of molecules that act as “security brakes” at multiple stages of the immune response. These regulations are important to prevent the destruction of tissues caused by inappropriate and/or disproportionate responses to invading pathogens. Cancer cells exploit such inhibitory immune checkpoints (ICPs) to escape destructive tumor-specific immune responses, which is unfavorable for the patients’ outcome.

In 2018, cancer caused 9.6 million deaths and was diagnosed in 18.1 million patients worldwide [1]. Cancer treatments focusing on reinvigorating exhausted tumor-specific immune cells significantly improved the survival of patients. In particular, the anti-tumor effects observed after monoclonal antibody (mAb)-mediated the blockade of the ICPs; cytotoxic T lymphocyte associated protein-4 (CTLA-4, CD152), programmed death-1 (PD-1, CD279) and its ligand PD-L1 (CD274, B7-H1), revolutionized the field of immuno-oncology. Therapeutic success was achieved in a fraction of patients and provided clinical evidence that corroborated the preclinical finding that ICPs exert inhibitory effects on immune cells that try to eradicate cancer cells. However, the response frequencies to these widely used ICP-blocking mAbs are suboptimal as a consequence of, amongst others, tumor resistance, an absence of tumor-infiltrating lymphocytes (TILs), and the presence of inhibitory myeloid cells [2]. Moreover, the occurrence of immune-related adverse events (irAEs) has been a reason to discontinue the use of ICP-blocking mAbs. Taken together, researchers are continuously untangling the biology of inhibitory receptors to increase response rates and to prevent irAEs.

The list of inhibitory ICPs that negatively regulate anti-tumor immune responses is growing. Among these next-generation ICPs, lymphocyte activating gene-3 (LAG-3, CD233) has emerged as an eminent target in the development of cancer treatment and holds substantial prognostic value. With as many as 15 different compounds targeting LAG-3 under (pre)clinical evaluation, it is the most widely studied ICP next to CTLA-4 and PD-1/PD-L1. LAG-3, also known as CD223, is expressed on numerous immune cell types, where its exact mechanism of action is yet to be fully discovered. However, it is certain that LAG-3 shows a remarkable synergy with PD-1 in promoting immune escape of cancer cells [3,4,5,6]. Notably, the simultaneous blockade of LAG-3 and PD-1 has shown striking clinical results in melanoma patients that were not responding well to initial PD-1 or PD-L1 monotherapy [7,8].

The growing list of inhibitory ICPs brings forth the challenge of defining which patients are likely to benefit from ICP therapy and which inhibitory ICP(s) prevail(s) in the patients’ tumors and therefore should be targeted. Presently, patients are selected for treatment with ICP-blocking mAbs, depending on the level of inhibitory ICP expression in the tumor microenvironment (TME), which is determined using immunohistochemistry (IHC). The static picture created by the IHC of a tumor biopsy does not take in account the heterogeneously distributed expression of ICPs. The latter can explain the fact that “non-expressors” do respond to ICP-blocking therapies [9]. Contrarily, some patients with IHC samples that show a clear expression of ICPs in the TME are observed not to react to ICP-blocking mAbs [10,11]. This can be due to amongst others, the compensatory expression of other ICPs [9,12,13]. Recent studies explored the field of molecular imaging as a more suited alternative for the non-invasive detection of ICPs in cancer patients. Molecular imaging allows the whole body visualization of prognostic or predictive markers. It can be performed non-invasively and repetitively, which allows quantifying and tracking of the dynamic evolution of expression patterns.

The aim of this review is to provide an overview of the biology of LAG-3 in the context of cancer as well as to provide an overview on the strategies used to detect and block LAG-3.

## 2. The Receptor LAG-3 and Its Interaction Partners

**Figure 1:**
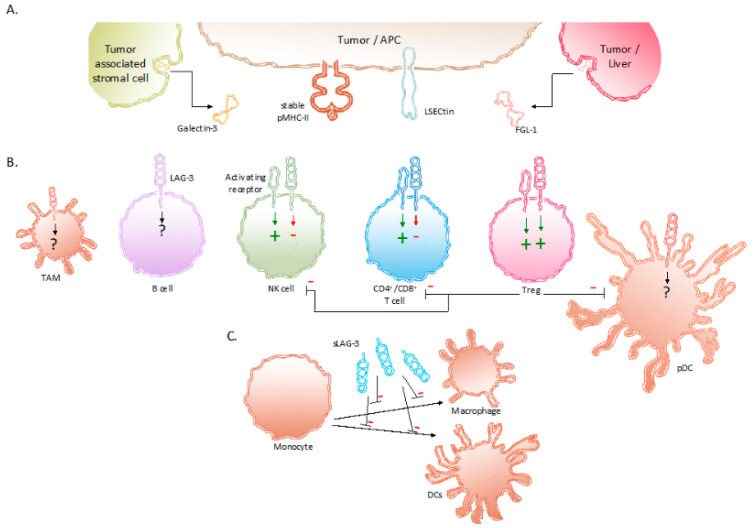
Expression of LAG-3 and its ligands in the TME. (**A**) Binding partners reported to associate with LAG-3 are stable pMHC-II complexes, Gal-3, LSECtin and FGL-1. These LAG-3 ligands are expressed on APCs, tumor cells and tumor-associated stromal cells. (**B**) Expression of LAG-3 on different immune cells and its influence on their effector functions. (**C**) sLAG-3 inhibiting the differentiation of monocytes towards macrophages or DCs. Abbreviations: APC, antigen-presenting cell; FGL-1, fibrinogen-like protein 1; Gal-3, Galectin-3; LSECtin, liver sinusoidal endothelial cell lectin; NK, natural killer; pDC, plasmacytoid dendritic cell; pMHC-II, peptide major histocompatibility complex class II; sLAG-3, soluble LAG-3; TAM, tumor-associated macrophage; TME, tumor microenvironment; Treg, regulatory T cell.

LAG-3, also called CD223, was identified in 1990 as a receptor that was expressed on a natural killer (NK) cell line cultured with interleukin (IL)-2 [14]. A close relationship with the cell surface protein CD4 was shown in terms of location and organization of its coding region as well as in terms of amino acid sequence and protein structure. The *LAG-3* gene is located on the distal portion of the short arm of human chromosome 12 (12p13.31), which is adjacent to the coding region for CD4, and contains eight exons [15]. The conservation of *LAG-3* among species is shown by its 70% and 78% homology with murine [14] and pig [16] *LAG-3*, respectively. The protein LAG-3 is 503 amino acids large, weighs 70 kDa, and is a type I transmembrane protein that contains four extracellular Immunoglobulin (Ig)-like domains termed domain 1 to 4. Approximately 20% of its amino acid sequence is identical to the CD4 protein, which is mostly pronounced in the extracellular region [14,17]. In contrast, the intracellular region of CD4 and LAG-3 lack homology, suggesting different functions. Due to the extracellular similarity, CD4 and LAG-3 share the same ligand, i.e., major histocompatibility complex class II (MHC-II) proteins. In LAG-3, a 30 amino acid long “extra loop” in domain 1 has been reported to engage MHC-II [18,19]. Notably, LAG-3 does not universally recognize MHC-II/peptide (pMHC-II) complexes on the surface of antigen-presenting cells (APCs). LAG-3 has been shown to selectively bind pMHC-II complexes that are considered stable after H2-DM has replaced the class-II-associated invariant chain peptide or unstable peptides by peptides with high affinity for the peptide-binding groove of MHC-II. LAG-3 is able to bind these stable pMHC-II complexes with a stronger affinity than CD4 [15,18,19,20,21]. Several other ligands interact with LAG-3, including Galectin-3 (Gal-3), liver sinusoidal endothelial cell lectin (LSECtin, CLEC4G) and fibrinogen like protein 1 (FGL-1) (Figure 1).

LSECtin is mainly expressed in the liver and has been found on the surface of tumor cells, such as human melanoma cells, as an engendered mechanism of immune escape [22,23]. Gal-3 is a soluble molecule secreted by a variety of tumor cells and tumor-associated stromal cells. It interacts with LAG-3 and was observed to reduce the frequency of CD8^+^ T cells producing interferon gamma (IFN-γ) in the TME [24,25]. The fourth ligand of LAG-3, FGL-1, has been identified more recently. FGL-1 is produced and secreted by tumor cells and hepatocytes. Preclinical work showed that FGL-1 reduced the production of IL-2 by a T-cell line, when interacting with LAG-3 on the T-cell surface [26].

## 3. Expression of LAG-3 and Its Regulation in Tumor-Associated Immune Cells

As illustrated in Figure 1, the expression of LAG-3 in the TME has been observed on TILs [27], in particular CD4^+^ and CD8^+^ T cells [14,28], including regulatory T cells (Tregs) [29]; NKT cells [30]; B cells [31]; NK cells [32] as well as on plasmacytoid dendritic cells (pDCs) [33,34] and tumor-associated macrophages (TAMs) [35,36].

It has been shown that LAG-3 expression on CD4^+^ and CD8^+^ T cells, even NKT cells, is induced upon continued antigen stimulation further stimulated through exposure to IFN-γ, IL-2, IL-7, and/or IL-12 [20,30,37,38]. Several transcriptional regulators have been implicated in the regulation of LAG-3 expression and T-cell exhaustion, which is the state of most TILs. These are thymocyte selection-associated high mobility group box protein (TOX), nuclear factor of activated T cells (NFAT), and nuclear receptor subfamily 4, group A (NR4A) [39,40,41,42,43,44]. LAG-3 expression is further regulated through its trafficking in cells [45]. In addition, proteolytic cleavage by ADAM10 and ADAM17, two metalloproteases, regulates LAG-3 levels on the T-cell surface, allowing T-cell activation in the initiation phase of the immune response [46]. However, sustained antigen stimulation forces LAG-3 expression on the cell surface, leading to the loss of T-cell effector functions and the development of an exhausted state.

Several Treg-populations have been characterized by high and constitutive expression of LAG-3 [47,48,49]. These include CD4^+^ Foxp3^+^ Tregs, IL-10 producing Tr1 cells as well as IL-10 and transforming growth factor beta (TGF-β)3 producing CD4^+^ Foxp3^-^ Tregs. It has been hypothesized that this constitutive LAG-3 expression is due to continuous TCR signaling by self-antigens, which is recognized by these Treg-populations, implicating the same transcriptional regulators, as described for CD4^+^ and CD8^+^ T cells with an effector function [50]. Moreover, early growth response gene 2 (EGR2) has been described as a critical regulator of LAG-3 expression in CD4^+^ Foxp3^-^ Tregs [29].

The expression of LAG-3 on B cells has not been elucidated on the molecular level. However, it has been shown to depend on activated T cells [31]. B cells have been shown to express LAG-3 when cultured with anti-CD3 antibody activated T cells, however, not when stimulated with antibodies that bind the B-cell receptor and CD40, mimicking interaction with CD4^+^ T_H_ cells. Further analysis of the mechanisms leading to LAG-3 acquisition on the B-cell surface has revealed the need for a soluble factor to induce endogenous LAG-3 expression in B cells with IL-6 being a good candidate. It has been further shown using LAG-3 knock-out cells that LAG-3 detected on B cells upon co-culture with activated T cells can further be acquired through the absorption of LAG-3 from activated T cells.

The regulation of LAG-3 expression on cells of the innate immune system, including NK cells, pDCs, and macrophages, has yet to be elucidated. Although some hints to the signals that can induce LAG-3 expression on NK cells and pDCs are available, the molecular mechanisms are not yet described. LAG-3 expression has been induced on NK cells using IL-12 [51,52,53]. Moreover, a subpopulation of NK cells, more specifically NKG2C^+^ NK cells, have been shown to express LAG-3 in response to interaction with an NKG2C agonist and IL-15 [32]. These cells further showed increased PD-1 expression, suggesting that LAG-3 and PD-1 expression coincide in this NK cell population, similar as in T cells. LAG-3 expression has been shown on a subset of pDCs in healthy individuals as well as on pDCs in melanoma patients with a clear enrichment in LAG-3^+^ pDCs in melanoma-invaded lymph nodes and in cutaneous melanoma metastasis. Moreover, it was shown that stimuli such as IL-3 and CpG DNA could stimulate LAG-3 expression on these cells [34].

## 4. Signaling Induced by LAG-3 and Its Net Effect

Thirty years after its discovery, the exact signaling pathway of LAG-3 remains unknown. Compared to other ICPs such as PD-1 and CTLA-4, LAG-3 does not have immunoreceptor tyrosine-based inhibitory motifs (ITIM) present in its cytoplasmatic tail. It has been shown that the inhibitory effect of LAG-3 is not caused by competitive pMHC-II binding with CD4 [21,28]. Instead, Workman et al. reported an intracellular “KIEELE” sequence in the intracellular part of LAG-3 on CD4^+^ T cells that is necessary to transduce distinctive yet undetermined inhibitory signals [19]. This finding was recently contradicted in a study reporting that LAG-3 elicits inhibitory mechanisms through an F*XX*L motif present in the membrane-proximal regions in cooperation with a C-terminal E*X* repeat [54]. The latter is found to interact with a so-called LAG-3-associated protein (LAP) [55]. The inhibitory effect of LAG-3 was lost upon deletion of the E*X* repeat and when mutations were induced in the FXXL sequence [54]. Although the molecular mechanisms exploited by LAG-3 to install inhibitory signals are slowly being uncovered, there is still much to learn about LAG-3 and how its atypical cytoplasmic motifs interact with cytoplasmic signaling proteins.

The biological activity of LAG-3 is most intriguing. LAG-3 has been shown to transduce inhibitory signals on activated CD8^+^ T cells even though the activation of CD8^+^ T cells is not driven by peptide presentation in MHC-II. Nonetheless, the inhibition of CD8^+^ T-cell activation has been shown to be induced by APCs that express high amounts of pMHC-II in addition to pMHC-I [21]. Moreover, together with the co-expression of PD-1, the anti-tumor immunity of CD8^+^ T cells could be abolished through the interaction with LAG-3’s other ligands i.e., LSECtin, FGL-1, and Gal-3 found in the TME [23,24,26,56]. Furthermore, Tregs present in the TME are renowned to weaken cancer-specific immune responses through the downregulation of inflammatory cytokines and the upregulation of suppressor activity. The role of LAG-3 has been shown to be essential in supporting Treg activity [47]. Research in non-small-cell lung cancer (NSCLC) patients show elevated LAG-3 expression on Tregs residing in the tumor compared to Tregs found in peripheral blood and normal tissues [57,58]. The surface expression of LAG-3 on Tregs has been shown to increase the secretion of immune suppressive cytokines, such as IL-10 and TGF-β [57,58]. These cytokines elicit inhibitory effects on the activity of CD8^+^ T cells, NK cells, and DCs. Furthermore, Tregs directly inhibit pDCs through LAG-3–pMHC-II interactions. These have been shown to initiate suppressive pathways, which hamper the proliferation and maturation of DCs [59]. Moreover, of all DC subsets, pDCs constitutively express LAG-3, which in turn negatively regulates their activation, intrinsic physiology, and extrinsic interplay with T cells [33]. Consequently, tumor-infiltrating pDCs expressing LAG-3 have been shown to contribute to an anti-inflammatory environment in melanoma patients [34]. The direct role of LAG-3 expressed on activated NK cells is still not fully understood. Although upregulated on human NK cells in response to IL-12, the blockade of LAG-3 on NK cells has shown no specific influence on their functionality [51,52,53]. However, on NKT cells expressing both NK receptors and T cells receptors, LAG-3 has shown to downregulate their proliferation [30]. As mentioned above, the expression of LAG-3 on B cells has been shown to be T-cell dependent [31]. More recently, Lino et al. identified a plasma B cell subset, selectively expressing LAG-3, with immune suppressing activity through the production of IL-10 [60]. Additionally, digital spatial protein analysis demonstrated the expression of LAG-3 in TAMs [35]. Although its role is still not fully understood, it can be speculated that LAG-3 expression on TAMs contributes to their tumor-promoting function, as suggested by the association of co-expression of CD163 and LAG-3 with poor clinicopathological indexes in melanoma [36] and metastatic ovarian cancer [61]. As mentioned above, LAG-3 can be cleaved from the cell surface by ADAM10 and ADAM17 and form soluble LAG-3 (sLAG-3) [46]. sLAG-3 regulates immune responses in the TME and periphery, for example by inhibiting the differentiation of monocytes to macrophages or DCs (Figure 1) [62,63,64].

## 5. The Prognostic Value of LAG3

LAG-3 is known to promote tumor escape through an induction of immunosuppression [21,65,66,67]. Consequently, the presence of LAG-3 on tumor-infiltrating immune cells has been described to be associated with poor prognosis and tumor progression. Individual studies reported this observation for various tumor types, including renal cell carcinoma [68], gastric cancer [69], bladder cancer [70], colorectal cancer [71], chronic lymphocytic leukemia [72], acute myeloid leukemia [73], follicular lymphoma [74], hepatocellular carcinoma [75,76], NSCLC [58], head and neck squamous cell carcinoma [77], esophageal squamous cell carcinoma [78], and diffuse large B cell lymphoma [79]. These studies suggested a contribution of LAG-3 to immune escape by the tumor, similarly to PD-1.

Paradoxically, some clinical reports indicated a favorable outcome of cancer patients when LAG-3 expression is observed on tumor-infiltrating immune cells. This is the case for breast cancer [80,81], esophageal adenocarcinoma [82], and advanced gastric cancer patients treated with PD-1 blocking mAbs [83]. Here, LAG-3 expression could be considered a marker for T-cell activation. Moreover, Saleh et al. [84] recently performed a meta-analysis on the prognostic value of LAG-3 expression in a variety of tumor types, observing that LAG-3 expression was associated with a better overall survival (OS), particularly in patients with early stage disease [84]. This might be explained by the fact that LAG-3 is expressed on activated immune cells, such as CD8^+^ T cells, and therefore might reflect infiltration of the tumor and initial tumor control by these immune cells [81,85]. Similarly, the expression of ICP PD-L1 and CTLA-4 have been previously associated with improved tumor outcomes [86,87,88].

The contradictory observations made about the prognostic value of LAG-3 expression in cancer patients could be associated to the difference in evaluated tumor type. However, more research is required to confirm if a tumor-specific prognostic value of LAG-3 really exists.

## 6. Immunohistochemistry and Imaging of the Immune Checkpoint LAG-3

**Figure 2:**
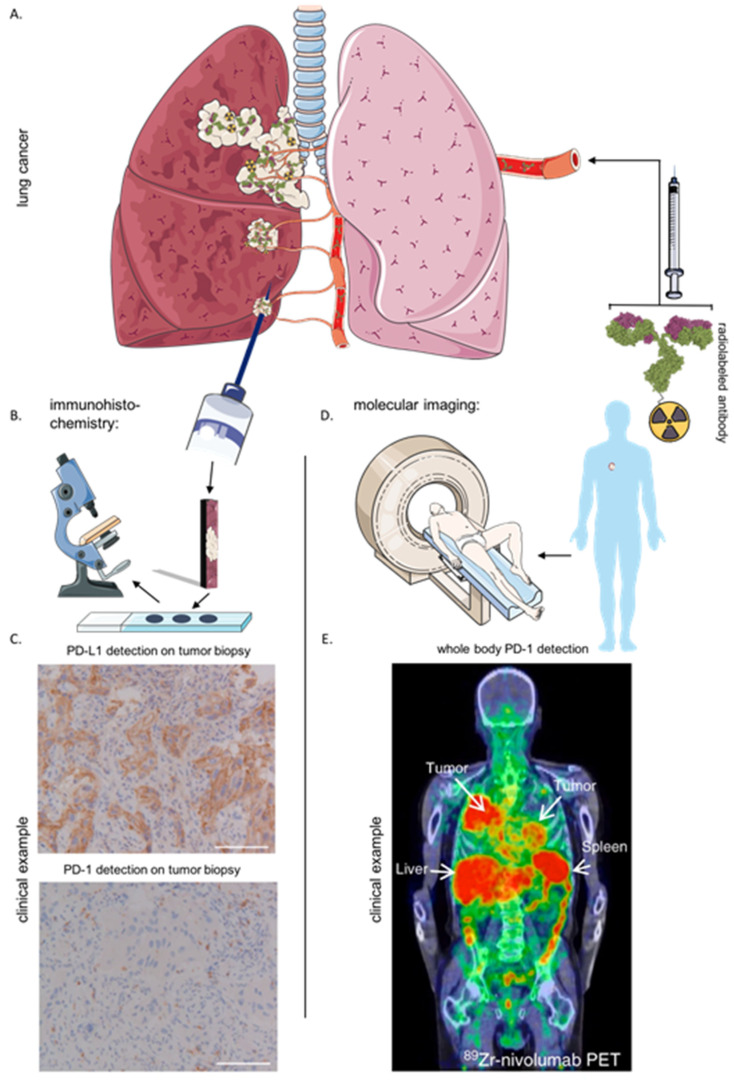
Comparison of IHC with molecular imaging for the detection of immune checkpoints. (**A**) Illustration of lung cancer that has metastasized to other regions. (**B**) Schematic representation of acquiring a biopsy of a tumor lesion and evaluation of biomarker expression using IHC. (**C**) Clinical example of PD-1/PD-L1 detection using IHC on a biopsy of NSCLC patient (adapted form Niemeijer et al. 2018 [91]). (**D**) Schematic representation of the intravenous injection of radiolabeled targeting moieties and the medical imaging devices used to visualize the tracer throughout the patient’s body. (**E**) Clinical example of PD-1 detection using molecular imaging in a NSCLC patient with radiolabeled antibody Nivolumab (adapted form Niemeijer et al. 2018 [91]). Abbreviations: IHC, immunohistochemistry; NSCLC, non-small cell lung cancer; PD-1, programmed death-1; PD-L1, programmed death-ligand 1; ^89^Zr, Zirconium^89^; PET, positron emission tomography.

The expression of LAG-3 is mostly studied using next-generation sequencing or IHC. The latter comprises the need to acquire invasive biopsies and cannot be considered a representative image of the heterogeneous distribution of ICPs within the TME. More importantly, the dynamic expression of ICPs and its role outside the TME can cause misinterpretation when IHC is used as a selection tool to predict therapy outcome. These limitations are summarized elsewhere more extensively [89]. In contrast to IHC, the use of molecular imaging to non-invasively detect molecules throughout the patient’s body has proven to be a more effective way to correlate therapy outcome [90,91]. As illustrated in Figure 2, IHC generates a static picture of a selected area of one tumor lesion compared to the possibility of nuclear imaging to generate whole body target distribution profiles. Moreover, nuclear imaging can be performed repetitively, regardless of the tumors’ location. For example, mAbs targeting PD-1 or PD-L1 were radiolabeled with isotopes for positron emission tomography (PET) and intravenously injected in cancer patients. After scanning the patients, molecular imaging was proven to be more accurate in predicting response to ICP therapy compared to the use of IHC [90,91]. Moreover, recent preclinical studies have evaluated the use of nanobodies as targeting moiety for radionuclide imaging, which provided high-contrast images within 2 h after injection, enabling its straightforward implementation in clinical routine [9,89,92].

In view of LAG-3 detection, we report two recent preclinical studies that evaluated molecular imaging for non-invasive mapping of LAG-3 using a mAb or a nanobody. Kelly et al. described, in a congress report, the use of a Zirconium^89^ (^89^Zr) labeled antibody directed against human LAG-3 [93]. Mice, subcutaneously implanted with a mix of human cancer and immune cells, were subjected to PET scans after administration of the radiolabeled antibody. ^89^Zr-DFO-REGN3767 specifically accumulated in the tumor, spleen, and axillary lymph nodes 6 days after administration to the mice. The visualization of radioactivity suggests the presence of LAG-3 expression on cells residing in these tissues. However, due to poor penetration capacity and slow clearance of free tracer, the radiolabeled antibody needed up to 6 days to map LAG-3 with high contrast, and thus, radioisotopes with longer half-lives were required. When translated to a clinical setting, the injected patient will have to wait a substantial amount of time before a scan can be performed. Moreover, during this “incubation” time the patient is exposed to a considerable amount of radioactivity [94]. Therefore, research has focused on using smaller binding moieties with better in vivo penetration capacity to non-invasively detect LAG-3. We reported a nanobody that binds mouse LAG-3 [95]. Nanobodies are derived from the antigen-binding domain of heavy-chain only antibodies found in among other camelids [89]. A feature of nanobodies is that they are around ten times smaller in size than mAbs. As a consequence, nanobodies can easily penetrate dense tissues such as tumors [96,97]. Other advantageous traits include high specificity, high affinity, high (thermo)stability, low off-target accumulation, and good solubility [98]. Additionally, nanobodies have been described that are able to transmigrate through the blood–brain barrier [99,100,101]. Preclinical evaluation in mice showed the feasibility of detecting LAG-3 as soon as one hour after injection with radiolabeled nanobodies [95]. Moreover, due to its small size, unbound radiolabeled nanobodies are quickly filtered from the blood by the kidneys to end up in the urine, avoiding long exposure to the decaying isotope [102]. This would mean that a patient could be scanned quickly after administration and, depending on the target, enrolled for appropriate ICP therapy.

## 7. Clinical Evaluation of LAG-3 Targeted Treatment in Cancer

Since its discovery, many preclinical studies confirmed that the blockade of LAG-3 could support anti-cancer immune responses, leading to a significant delay in tumor growth compared to control conditions [3,4,103,104,105,106,107,108,109]. Notwithstanding these preclinical data, more recent clinical research shows that the blockade of LAG-3 alone might not be an ideal treatment strategy. This might be explained by the ability of cancer cells to evade anti-tumor immune responses through a myriad of molecules. However, our lack of in-depth understanding of the mechanisms exploited by LAG-3 to suppress immune activity makes it difficult to explain the limited effect of LAG-3 blockade when used as a monotherapy. Notably, LAG-3 therapy is shown to be more effective when combined with other anti-cancer treatments. For example, Grosso et al. observed an increased number of tumor-infiltrating, activated CD8^+^ T cells when LAG-3 blockade was combined with tumor-associated antigen vaccination [103]. Moreover, co-blockade or genetic deletion of LAG-3 and PD-1 has shown strong therapeutic effects in various mouse tumor models [3,104,105,109]. Taken together, these observations encouraged pharmaceutical companies to implement LAG-3 as a promising therapeutic target in their pipeline.

**Figure 3:**
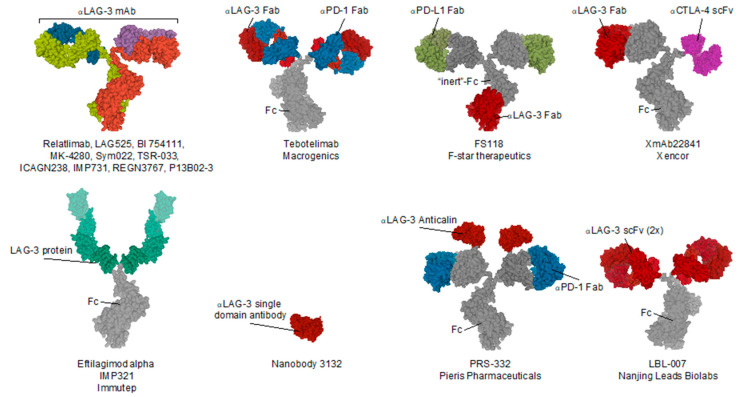
Schematic illustration of the structure and composition of LAG-3 targeting compounds evaluated as anti-cancer therapeutic and/or molecular imaging tracer. Abbreviations: Fab, antigen binding fragment; Fc, fragment crystallizable; scFv, single-chain variable fragment; α-LAG-3, anti-LAG-3.

As of today, 15 different LAG-3 blocking compounds have been evaluated in preclinical or clinical setting and purposed for the treatment of cancer. Table 1 summarizes these compounds and highlights their use for treating different cancer types as well as the clinical phase reached and the combination strategy that the developers have opted for. Most clinical trials are combining LAG-3 blockade with PD-1 or PD-L1 blocking compounds. Moreover, companies such as Fstar and Macrogenics are developing a mAb that simulatenously targets LAG-3 and PD-L1, or PD-1 respectively [110,111]. A schematic illustration of these compounds can be found in Figure 3.

Although many preclinical studies show therapeutic synergy when combining LAG-3 and PD-1 blockade, only a few reported on its clinical efficacy. As an example, Relatlimab, a mAb targeting LAG-3 from BMS, is being evaluated in combination with Nivolumab (anti-PD-1) for its safety and efficacy in patients with advanced melanoma refractory to prior anti-PD-1 or anti-PD-L1 blocking treatments [112]. Based on the data reported during the 2017 ESMO congress, they observed an objective response in seven out of 61 patients (11%) [8]. Moreover, prior to treatment, LAG-3 expression on tumor-associated immune cells was evaluated using IHC on a tumor biopsy. Retrospective analysis has demonstrated a response rate of 18% or 5% when efficacy is evaluated in the LAG-3^+^ group (>1% LAG-3 expression) versus LAG-3^−^ group (<1% LAG-3 expression), respectively. Additionally, an increase in response rate to 27% has been demonstrated in patient cohorts that scored LAG-3^+^ and PD-L1^+^ (>1% PD-L1 expression). These results highlight the importance of patient stratification to avoid the treatment of patients that are less likely to respond. Furthermore, Novartis/Prima Biomed combined Spartalizumab (anti-PD-1) with their LAG-3 targeting antibody LAG525. Durable responses were shown in 10 out of 121 patients (9.9%) with various types of solid tumors [113].

Several first-in-class bispecific antibodies are undergoing phase I clinical trials. Macrogenics’ Tebotelimab is a mAb harboring antigen binding fragments (Fab) targeting both PD-1 and LAG-3 [111]. Similarly, Pieris’ compound PRS-332 harbors two Fab regions solely targeting PD-1 and two anticalins, which are engineered lipocalins that target LAG-3 [114]. Moreover, antibody FS118 from F-star therapeutics harbors an antigen-binding fragment targeting LAG-3 in its constant region, leaving the Fabs as PD-L1 targeting domains [110]. Xencors XmAb22841 is an antibody with one Fab targeting LAG-3, and the other Fab is replaced by a single-chain variable fragment (scFv) targeting CTLA-4 [115]. Future reports on these constructs are awaited to gather insight on their actual therapeutic efficacy.

In addition to the use of antibodies, other LAG-3-targeting agents have been evaluated for the treatment of cancer. Molecule IMP321 is a fusion protein consisting of extracellular LAG-3 regions coupled to an Fc-region of IgG [116]. IMP321 was previously shown to activate APCs through interaction with MHC-II, which is present on their membrane [117,118]. Although preclinical work was able to show an increase in IL-12 and tumor necrosis alpha (TNF-α) production after administration of IMP321, its clinical efficacy was only minimal even when combined with other types of treatments [119,120,121].

We expect that all studies, once completed, will give more insight in the biological properties of LAG-3, which will ultimately help rationalize the use of these LAG-3 targeting agents for the treatment of cancer patients.

## 8. Conclusions and Perspectives

Although the biological processes of LAG-3 are yet to be fully discovered, more than 15 different moieties targeting this ICP have been developed. Moreover, it has been suggested that accurate patient stratification could help select patients that are more likely to respond. Consequently, different research groups are untangling the advantages of using molecular imaging to detect whole body target expression over the use of IHC. Although not yet proved for the ICP LAG-3 in particular, the advantage of using molecular imaging over IHC has already been demonstrated for the ICP PD-L1 [90,91]. However, the visualization of ICPs using radiolabeled antibodies is not practical. Therefore, smaller sized nanobodies with better penetration capacities and more rapid clearance are expected to be an ideal alternative. Combinatory treatments that incorporate the blockade of LAG-3 are viewed as a promising approach to improve current immunotherapies. Indeed, preclinical work in the field of immuno-oncology indicates that combination immunotherapies that include LAG-3 blockade could have synergic effects on anti-cancer immune responses. Additionally, early clinical data of BMS’s LAG-3 targeting antibody Relatlimab showed an improved OS of cancer patients when combined with the PD-1 blocking mAb Nivolumab. These preclinical and clinical results catalyzed an excitement for LAG-3 blockade that is spilling into several pharmaceutical companies, hence encouraging the further development of moieties that bind and block LAG-3.

## Figures and Tables

**Table 1:** Table summarizing the 15 compounds under development for blockade of LAG-3.

Name (Code)	Company	Format	Tumor Type	Study Phase	National Clinical Trial nr.	Combined with
PRS-332	Pieris Pharmaceuticals	LAG-3xPD-1 bispecific fusion protein	Solid	preclinical	/	/
P13B02-3	Agenus	IgG1	Solid	preclinical	/	(PD-1)
LBL-007	Nanjing Leads Biolabs	scFv—IgG4 fusion	Solid	preclinical	/	(mouse PD-1) BE0146 BioXcell
Eftilagimod alpha (IMP321)	Immutep	LAG-3 IgG1 Fc fusion protein	Solid, NSCLC, HNSCC, Breast, Melanoma	I & II	03252938, 00351949, 00349934, 02676869, 03625323, 02614833	(PD-L1) Avelumab antibody, (PD-1) Pembrolizumab antibody
LAG525 (IMP701)	Novartis/Prima Biomed	Humanized IgG4	Solid (advanced)	I/II & II	02460224, 03365791	(PD-1) Spartalizumab antibody
MK-4280	Merck Sharp & Dohme	Humanized IgG4	Hematological	I/II	03598608	(PD-1) pembrolizumab (MK-3475) antibody
REGN3767	Regeneron Pharmaceuticals	hinge-stabilized IgG4	Solid (advanced)	I	03005782	(PD-1) Cemiplimab (REGN2810)
Relatlimab (BMS-986016)	Bristol-Myers Squibb	Human IgG4	Melanoma, Hematological, Glioblastoma, Kidney, Lungs, Colon	I, I/Iia, II & II/III	02658981, 03335540, 02966548, 02061761, 01968109, 03459222, 02488759, 02996110, 02935634, 02750514, 02060188, 03470922	(PD-1) Nivolumab (BMS-936558) antibody
BI 754111	Boehringer Ingelheim	Humanized IgG4	HN, NSCLC, Solid (metatstatic)	I & II	03964233, 03697304	(PD-1) BI 754091 antibody, (VEGF/Ang2) BI 836880 bispecific nanobody
FS118	F-star Therapeutics	LAG-3 x PD-L1 tetravalent bispecific IgG1 antibody	Solid (advanced & metatstatic)	I	03440437	/
Tebotelimab (MGD013)	MacroGenics	LAG-3 × PD-1 bispecific IgG4k antibody	Solid, Cholangiocarcinoma, Liver, Gastric (HER-2^+^), Breast (HER-2^+^), Oesophageal, Haematological	I	03219268	(HER2) Margetuximab
TSR-033	Tesaro	Humanized IgG4	Solid (advanced)	I	03250832	(Tim-3) TSR-022 antibody, (PD-1) dostarlimab (TSR-042) antibody, (VEGF-a) Bevacizumab antibody, mFOLFOX6, FOLFIRI
INCAGN2385	Incyte	t.b.a.	Solid, Melanoma	I	04370704	/
Sym022	Symphogen	Human Fc-inert	Solid, Lymphoma	I	03311412, 03489369	(PD-1) Sym021 antibody, (Tim-3) Sym023 antibody
XmAb22841	Xencor	LAG-3 x CTLA-4 bispecific Fc-inert antibody	Solid	I	03849469	(PD-1) Pembrolizumab antibody

Abbreviations: Ang2, angiotensin 2; CTLA-4, cytotoxic T lymphocyte-associated protein 4; Fc, fragment crystallizable; HER, human epidermal growth factor receptor; HN, head and neck cancer; Ig, immunoglobulin; NSCLC, non-small-cell lung cancer; PD-1, programmed death 1; PD-L1, programmed death-ligand 1; scFv, single-chain variable fragment; t.b.a., to be announced; TIM-3, T-cell immunoglobulin and mucin domain-3; VEGF, vascular endothelial growth factor; nr.; number.

## Abbreviations

ICP	immune checkpoint
mAb	monoclonal antibody
CTLA-4	cytotoxic T lymphocyte associated protein-4
PD-1	programmed deat-1
TIL	tumor-infiltrating lymphocyte
irAEs	immune-related adverse events
LAG-3	lymphocyte activating gene-3
TME	tumor microenvironment
IHC	immunohistochemistry
Ig	immunoglobulin
MHC-II	major histocompatibility complex
pMHC-II	peptide loaded MHC-II
APC	antigen-presenting cell
Gal-3	galectin-3
LSECtin	liver sinusoidal endothelial cell lectin
FGL-1	fibrinogen like protein 1
IFN-γ	Interferon gamma
IL	Interleukin
Treg	regulatory T cell
NK	natural killer cell
pDC	plasmacytoid dendritic cell
DC	dendritic cell
TAM	tumor-associated macrophage
TOX	thymocyte selection-associated high mobility group box protein
NFAT	nuclear factor of activated T cells
NR4A	nuclear receptor subfamily 4, group A
TGF-β	transforming growth factor beta
EGR2	early growth response gene 2
ITIM	immunoreceptor tyrosine-based inhibitory motifs
LAP	LAG-3-associated protein
NSCLC	non-small-cell lung cancer
sLAG-3	soluble LAG-3
OS	overall survival
Zr^89^	Zirconium^89^
Fab	antigen binding fragment
scFv	single-chain variable fragment
TNF-α	tumor necrosis factor alpha
